# Repetitive Transcranial Magnetic Stimulation Induces Quantified Functional and Structural Changes in Subcortical Stroke: A Combined Arterial Spin Labeling Perfusion and Diffusion Tensor Imaging Study

**DOI:** 10.3389/fnhum.2022.829688

**Published:** 2022-04-06

**Authors:** Yu Jin, Xi Bai, Binghu Jiang, Zhiwei Guo, Qiwen Mu

**Affiliations:** ^1^Department of Radiology, Chengdu Second People’s Hospital, Chengdu, China; ^2^Department of Radiology, Langzhong People’s Hospital, Langzhong, China; ^3^Department of Radiology, Nanchong Central Hospital, Institute of Rehabilitation and Imaging of Brain Function, The Second Clinical Medical College of North Sichuan Medical College, Nanchong, China

**Keywords:** repetitive transcranial magnetic stimulation, arterial spin labeling, diffusion tensor imaging, subcortical stroke, motor function

## Abstract

**Purpose:**

To explore the changes of cerebral blood flow (CBF) and fractional anisotropy (FA) in stroke patients with motor dysfunction after repetitive transcranial magnetic stimulation (rTMS) treatment, and to better understand the role of rTMS on motor rehabilitation of subcortical stroke patients from the perfusion and structural level.

**Materials and Methods:**

In total, 23 first-episode acute ischemic stroke patients and sixteen healthy controls (HCs) were included. The patients were divided into the rTMS and sham group. The rehabilitation assessments and examination of perfusion and structural MRI were performed before and after rTMS therapy for each patient. Voxel-based analysis was used to detect the difference in CBF and FA among all three groups. The Pearson correlation analysis was conducted to evaluate the relationship between the CBF/FA value and the motor scales.

**Results:**

After rTMS, significantly increased CBF was found in the ipsilesional supplementary motor area, postcentral gyrus, precentral gyrus, pons, medulla oblongata, contralesional midbrain, superior cerebellar peduncle, and middle cerebellar peduncle compared to that during the prestimulation and in the sham group, these fasciculi comprise the cortex-pontine-cerebellum-cortex (CPC) loop. Besides, altered CBF in the ipsilesional precentral gyrus, postcentral gyrus, and pons was positively associated with the improved Fugl-Meyer assessment (FMA) scores. Significantly decreased FA was found in the contralesional precentral gyrus, increased FA was found in the ipsilesional postcentral gyrus, precentral gyrus, contralesional supplementary motor area, and bilateral cerebellum, these fasciculi comprise the corticospinal tract (CST). The change of FMA score was positively correlated with altered FA value in the ipsilesional postcentral gyrus and negatively correlated with altered FA value in the contralesional precentral gyrus.

**Conclusion:**

Our results suggested that rTMS could facilitate the motor recovery of stroke patients. High frequency could promote the improvement of functional activity of ipsilesional CPC loop and the recovery of the microstructure of CST.

## Introduction

Stroke is a major cause of mortality and disability and the main contributor to disability-adjusted life-years, such as disability, hemiparesis, aphasia, depression, and so on, both in developing or developed countries, which seriously affects people’s daily life (Katan and Luft, 2018). A previous study has reported that more than half of stroke patients died or became dependent after being discharged (Takashima et al., 2017). Reduced motor function leads to significant disability of stroke survivors, which affects their daily living and increases the burden on these patients and their families (Andrew et al., 2020). Therefore, early intervention may reduce the risk of poststroke mortality and disability. Although various treatment strategies have been used to improve motor function, such as pharmacotherapy, acupuncture (Li et al., 2015), and constraint-induced movement therapy (Kwakkel et al., 2015), poststroke movement disorders remain the current challenge.

Repetitive transcranial magnetic stimulation (rTMS), as a relatively new type of rehabilitation treatment, is a painless and non-invasive method for altering brain excitability. It has been widely used in treating depression, stroke, Parkinson’s disease, and others in recent years. According to the concept of interhemispheric competition, the cortical excitability changes within the two hemispheres after stroke. In stroke patients with motor dysfunction, the interhemispheric imbalance and reduced interinhibition have been reported both in animal and human studies (Lee et al., 2018). Rebalancing the cortical excitability between the hemispheres was crucial for the functional motor recovery of stroke patients (Siegel et al., 2016). Previous studies have demonstrated that high-frequency rTMS over the affected hemisphere could increase the excitability of the ipsilesional motor cortex, whereas low-frequency rTMS on the contralesional hemisphere could increase the excitability of the ipsilesional motor cortex as well by reducing excessive interhemispheric inhibition from the contralesional motor cortex (Du et al., 2019; Zhang et al., 2019; Xu and Sun, 2020).

Many previous studies, including a large sample size meta-analysis (Hsu et al., 2012), have confirmed the effectiveness of rTMS on stroke rehabilitation. However, its underlying therapeutic mechanism has not been fully elucidated. It may be closely related to its effects on Ca^2+^ dynamics, neurotransmitter release, neurotrophic factors, neuroendocrine system, glial network, neuroinflammation, oxidative response, and prevention of neuronal cell death (Cirillo et al., 2017). Along with the development of multimodality imaging technique, blood-oxygen-level-dependent (BOLD)-functional MRI, diffusion tensor imaging (DTI), perfusion, and so forth are widely used to investigate the brain functional changes of stroke patients. Therefore, these multimodal technologies also can effectively display the changes in brain structure and function caused by rTMS treatment.

Arterial spin labeling (ASL) is a non-invasive MRI technology that can quantify cerebral blood flow (CBF) without a contrast agent, and is sensitive when detecting perfusion changes (Zaharchuk et al., 2012; Thamm et al., 2019b). Currently, it has been widely used in the research of various diseases, namely, cerebrovascular disease, tumor, dementia, and so on (Ho, 2018). Previous studies focused on the perfusion changes in a different stages of stroke, and the results were abundant. Compared with the normal corresponding regions, the CBF values decreased in infarction lesions but increased significantly in adjacent areas in acute ischemic stroke (Guo et al., 2014). Other studies reported that CBF asymmetries detected by ASL are related to severity and outcome in acute stroke patients (Chalela et al., 2000), and higher CBF in the acute phase in the contralateral hemisphere predicts good outcome at day 90 (Thamm et al., 2019a). Takekawa et al. (2014) found that the perfusion measured by single-photon emission computed tomography improved after rTMS in chronic stroke, which was associated with upper limb motor function improvement. But to the best of our knowledge, no study has investigated the CBF changes measured by ASL after high-frequency rTMS treatment in acute stroke patients.

Diffusion tensor imaging plays an important role in evaluating the integrity of white matter tracts with fractional anisotropy (FA), which reflects the water diffusion information in all directions. The decrease of FA may be due to cell structural change, such as vascular alterations, demyelination, and gliosis (Brun and Englund, 1986; Englund, 1998). Structural changes determined the recovery of neuroplasticity and function after stroke. One study reported that the FA in the infarcted area was significantly lower than that in the unaffected side (Thomalla et al., 2004), suggesting that structural integrity is closely related to motor function. The corticospinal tract (CST) is the most affected. In subsequent experiments, the researchers found that increased FA values in ipsilesional CST were associated with better motor performance after poststroke intervention (Fan et al., 2015). Guo et al. (2016) also drew a similar conclusion, namely, that FA values of the affected CST and motor-related gray matter cortices increased significantly after high-frequency rTMS treatment. But Li et al. (2018) found that the FA value increased in contralesional CST after rTMS treatment. Therefore, the mechanism of poststroke recovery with DTI explanation still needs further research.

In this study, we aimed to identify the changes of the whole-brain CBF and FA, to explore the brain function and structural changes in pure subcortical stroke patients with motor dysfunction treated by high-frequency rTMS. It will provide objective evidence for the treatment mechanism of rTMS at both the functional and structural levels.

## Materials and Methods

### Participants

In total, 23 first-episode acute subcortical ischemic stroke patients and sixteen HCs were recruited, respectively, from the Department of Neurology of Nanchong Central Hospital and the Community. The patients’ inclusion criteria were (1) right-handedness, (2) adults (≥18 years) who suffered a first-ever ischemic stroke, (3) the lesion was located in unilateral subcortical, (4) hemiplegia, and (5) no history of neuropsychiatric diseases. The participants were excluded if they had any other neuropsychiatric diseases, audiovisual impairment, head injury, and relative or absolute contraindications of MRI. The lesions were mainly distributed in the corona radiate (6 patients), basal ganglia (13 patients), and pons (4 patients). The study was approved by the Ethics Committee of the Hospital, and all subjects were given informed consent before the experiment.

### Study Design and Intervention

Participants were randomly divided into the two groups (rTMS group and sham group). Each patient received a 10-day rTMS intervention (10 Hz, 30 trains, 50 pulses/train, and interval of 25 s) over the ipsilesional primary motor area beginning at about 4 days after stroke onset. The sham group received sham stimulation with the same parameters but the coil was positioned tangentially to the scalp of the stimulation target at a 90° angle from the mid-sagittal plane, so that no current flow was induced in the brain. The rTMS was performed by using a MagPro R30 magnetic stimulation device (MagVenture, Farum, Denmark) which was equipped with a figure-of-eight coil. The stimulation intensity was 90% resting motor threshold (RMT), and RMT was defined as the lowest stimulus intensity of rTMS which could elicit a motor-evoked potential at least 5 out of 10 trials (Groppa et al., 2012). The MRI scanning and rehabilitation assessments, namely, the Fugl–Meyer assessment (FMA), the National Institutes of Health Stroke Scale (NIHSS), and the Barthel Index (BI) were performed before and after rTMS therapy for each patient.

### Magnetic Resonance Imaging Data Acquisition

Magnetic resonance imaging examination was conducted before and after the stimulation in GE Signa HDxt 1.5 Tesla Scanner (General Electric Medical System, Milwaukee, WI, United States) using an 8-channel head coil with the same parameters. During the scanning, all the subjects were told to close their eyes, keep relaxed, and awake without any thinking. Noise reduction earplugs and sponge pads were given to protect their hearing and hold their heads still, respectively. The perfusion images were acquired using a three-dimensional-ASL (3D-ASL) sequence with the following parameters: repetition time (TR)/echo time (TE)/flip angle (FA) = 4,629 ms/10.5 ms/155°, field of view (FOV) = 24 cm × 24 cm, post-label delay (PLD) = 2,025 ms, slice number = 36, thickness = 4.0 mm, and gap = 0.0 mm. The DTI images were acquired by single-shot echo-planar imaging (EPI) sequence with the following parameters: TR/TE/flip angle = 8,500 ms/96 ms/90°, FOV = 24 cm × 24 cm, image matrix = 256 × 256, voxel sizes = 0.94 mm^3^ × 0.94 mm^3^ × 5.0 mm^3^, slice number = 32, thickness = 5.0 mm, and gap = 0.0 mm. The diffusion sensitivity coefficient *b* value equals to 1,000 s/mm^2^. The structural images were acquired using a 3D T1 fast spoiled gradient recalled (3D-T1FSPGR) sequence with the following parameters: TR/TE/flip angle = 9.1 ms/2.9 ms/20°, FOV = 24 cm × 24 cm, image matrix = 256 × 256, voxel sizes = 0.94 mm^3^ × 0.94 mm^3^ × 1.2 mm^3^, slice number = 124, and gap = 0.0 mm.

### Data Processing

Arterial spin labeling obtained a cerebral perfusion map by marking arterial blood water as an endogenous tracer, which included label image and control image. The CBF maps were gotten from GE FuncTool work station subsequently. The dcm2niigui software was used to convert the DICOM format of CBF into NIfTI format; then SPM8 (Statistical Parametric Mapping)^1^ /dpabi software was used to carry out spatial standardization, data normalization, smoothing, and flipping in sequence. The detailed steps were as follows. First, to eliminate the differences in the anatomical structure of the different subjects, the images of all the subjects were standardized to the Montreal Neurological Institute (MNI) spatial coordinate system. Second, the blood flow value of each part was divided by the mean value. Third, the images were smoothed by a Gaussian kernel with full width at half maximum (FWHM) of 8 mm to improve the signal-to-noise ratio (SNR). Fourth, the lesion was turned over to improve statistical power.

The DTI data were preprocessed with SPM8 software, too. At first, head motion correction. Then, using interpolation streamline propagation algorithm of the Diffusion Toolkit^2^ software to track the whole-brain fiber tracts of DTI images after preprocessing. The stop condition of tracking was FA > 0.15, tracking angle <35°, then the FA map was reconstructed. After that, spatial standardization and smooth of FA parameters were conducted. The SNR was improved by the FWHM of 8 mm.

### Statistical Analyses

The paired *t*-test was adopted to analyze the changes of CBF and FA after rTMS treatment. A two-sample *t*-test was used to evaluate the altered CBF, FA in stroke patients and HC, post-real and sham rTMS treatments. A statistically significant difference would be considered at *p* < 0.05. The results were presented on xjView software.

### Correlation Analyses

To explore the relationship between the CBF/FA changes in different brain regions and the improvement of motor function in pre- and post-rTMS treatments, the Pearson correlation coefficient was calculated between changed CBF, FA, and FMA score difference with age, gender as covariates. The significant threshold was set at *p* < 0.05.

## Results

### Basic Information of Subjects

The detailed basic information was shown in Table 1. There were no significant differences in age and time since stroke. Of 23 stroke patients, 15 received rTMS treatment and the other 8 received sham rTMS treatment. The mean age of the rTMS group, sham rTMS group, and HCs were 64.47 ± 9.39, 62.50 ± 7.54, and 63.06 ± 7.05 years, respectively. Compared to prestimulation, the motor function and daily living ability after postintervention in both the groups were all significantly improved according to the results of paired *t*-test. In addition, the increase in FMA was significantly greater in the rTMS group than sham group after treatment. All the patients completed the rTMS treatment without any side effects.

**TABLE 1:** Demographical clinical characteristics of all the included subjects.

		rTMS	Sham	Health control	*t*/χ^2^	*p*
Age (years)		64.47 ± 9.39	62.50 ± 7.54	63.06 ± 7.05	0.19	0.83
Gender (M/F)		15 (10/5)	8 (5/3)	16 (10/6)	0.07	0.96
Lesion location		Corona radiate (6 patients), basal ganglia (13 patients), pons (4 patients)				
Time since stroke (days)		5.00 ± 1.96	4.50 ± 2.07		0.562	0.583
FMA	Pre	35.27 ± 16.19	29.38 ± 14.23		0.901	0.381
	Post	47.87 ± 21.98^a,b^	31.38 ± 15.18^a^		2.111	0.048
NIHSS	Pre	7.07 ± 1.87	7.50 ± 1.51		–0.602	0.555
	Post	4.67 ± 1.76^a^	6.25 ± 1.75^a^		–2.061	0.058
BI	Pre	46.67 ± 18.19	35.00 ± 11.65		1.867	0.077
	Post	63.00 ± 19.25^a^	48.75 ± 14.08^a^		2.026	0.057

*FMA, Fugl-Meyer assessment; NIHSS, National Institutes of Health Stroke Scale; BI, Barthel Index; M, Male; F, Female.*

*^a^The significant difference between pre- and post-rTMS (p < 0.05).*

*^b^The significant difference between the rTMS group and sham group after treatment (p < 0.05). All data are shown as the mean ± SD.*

### Cerebral Blood Flow Changes Between Stroke Patients and Healthy Controls

**FIGURE 1:**
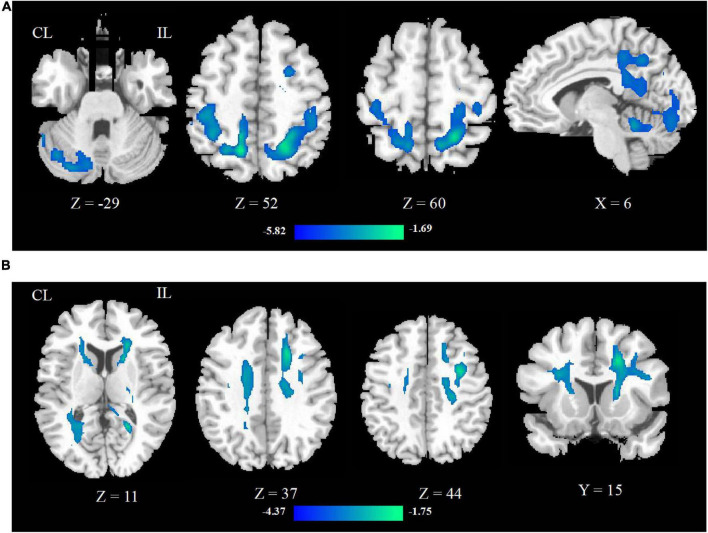
The CBF **(A)** and FA **(B)** changed between stroke patients and HCs. The blue color represents the decreased areas of CBF and FA values. CL, contralesional; IL, ipsilesional; HCs, healthy controls.

Compared with HCs, the CBF of stroke patients decreased in contralesional cerebellum, bilateral precentral and postcentral gyrus, superior parietal lobe, and contralesional posterior cingulate cortex. As shown in Figure 1A.

### Cerebral Blood Flow Changes Between Pre- and Post-rTMS Treatment

**FIGURE 2:**
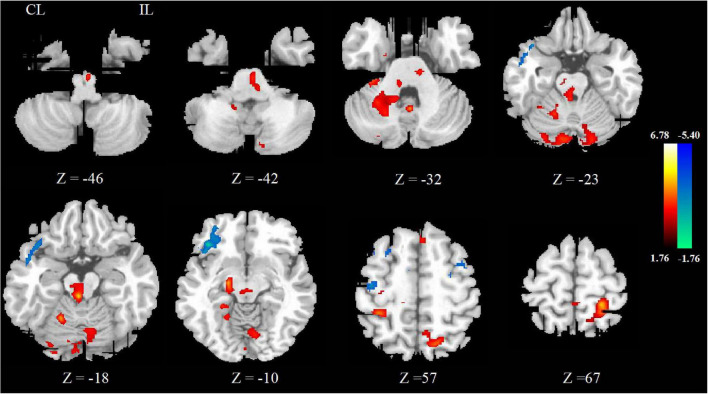
The change of CBF between pre- and post-rTMS treatment. The blue color represents the decreased areas of CBF value, and the red color represents the increased areas of CBF value. CL, contralesional; IL, ipsilesional.

After rTMS treatment, the CBF increased in ipsilesional postcentral gyrus, precentral gyrus and pons, contralesional midbrain, superior cerebellar peduncle, middle cerebellar peduncle, and ipsilesional medulla oblongata, and decreased in contralesional precentral gyrus. These mainly involved the ipsilesional cortex-pontine-cerebellum-cortex (CPC) pathway. The significant differences are given in Figure 2 and detailed information of significant clusters and peak voxels is shown in Table 2.

**TABLE 2:** Brain regions showing significantly different CBF between pre- and post-rTMS treatment.

Brain region	MNI coordinates (*x, y, z*)	*t* value	Voxel number
**Post > Pre**			
Postcentral _L	−22, −42, 66	4.55	198
Precentral _L			96
Superior cerebellar peduncle _R	8, −86, −28	4.05	642
Middle cerebellar peduncle _R			
Midbrain _R	2, −32, −20	5.02	482
Pons _L	−6, −32, −42	2.49	115
Medulla oblongata _L	−2, −22, −46	3.11	44
**Pre < Post**			
Precentral _R	40, −16, 56	−2.79	73

### Cerebral Blood Flow Changes Between Repetitive Transcranial Magnetic Stimulation Group and Sham Repetitive Transcranial Magnetic Stimulation Group

**FIGURE 3:**
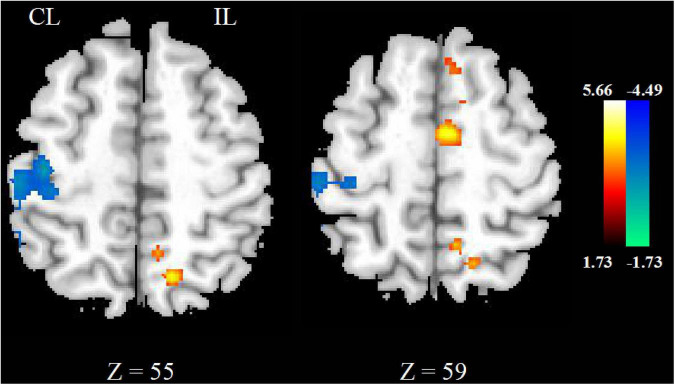
The differences of CBF between real and sham rTMS posttreatment.

The CBF increased in ipsilesional supplementary motor area (SMA) and decreased in contralesional precentral gyrus, postcentral gyrus in the real rTMS group, as shown in Figure 3 and Table 3.

**TABLE 3:** Brain regions showing significantly different CBF between real and sham rTMS posttreatment.

Brain region	MNI coordinates (*x, y, z*)	*t* value	Voxel number
**Post > Pre**			
Supplementary motor area _L	−4, −5, 58	4.05	646
**Post < Pre**			
Precentral _R	40, −20, 52	−4.09	239
Postcentral _R			398

### Fractional Anisotropy Changes Between Stroke Patients and Healthy Controls

Compared with HCs, the FA of stroke patients decreased in bilateral anterior limb of internal capsule, precentral and postcentral gyrus, superior parietal lobe, corona radiata, and ipsilesional precentral gyrus, as shown in Figure 1B.

### Fractional Anisotropy Changes Between Pre- and Post-rTMS Treatment

**FIGURE 4:**
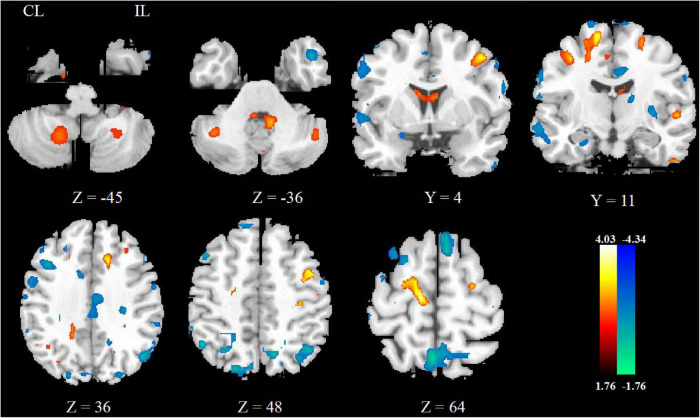
The FA changes between pre- and post-rTMS treatment.

After rTMS treatment, the FA increased in ipsilesional postcentral gyrus, precentral gyrus, contralesional SMA, and bilateral cerebellar hemisphere and decreased in contralesional precentral gyrus, as shown in Figure 4 and Table 4.

**TABLE 4:** Brain regions showing significantly different FA between pre- and post-rTMS treatment.

Brain region	MNI coordinates (*x, y, z*)	*t* value	Voxel number
**Post > Pre**			
Cerebellum _R	18, −58, −50	2.23	31
Cerebellum _L	−45, −58, −41	2.11	52
Postcentral _L	−36, −22, 43	2.82	31
Precentral _L	−42, −1, 49	3.61	41
Supplementary motor area _R	12, −19, 61	4.03	44
**Pre < Post**			
Precentral _R	57, −1, 34	−2.43	41

### Fractional Anisotropy Changes Between Repetitive Transcranial Magnetic Stimulation Group and Sham Repetitive Transcranial Magnetic Stimulation Group

**FIGURE 5:**
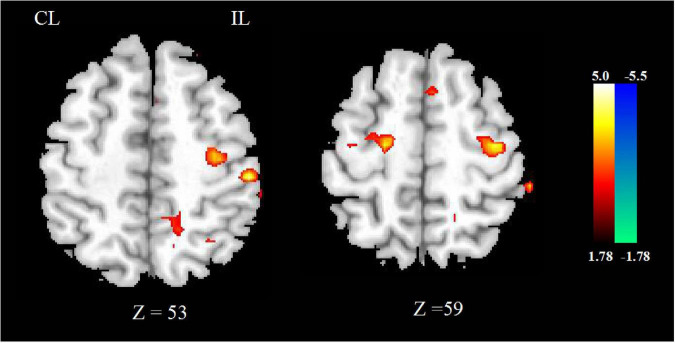
The FA changes between real and sham rTMS posttreatment.

The FA increased in bilateral precentral gyrus and ipsilesional postcentral gyrus in the real rTMS group other than the sham rTMS group, as shown in Figure 5 and Table 5.

**TABLE 5:** Brain regions showing significantly different FA between real and sham rTMS posttreatment.

Brain region	MNI coordinates (*x, y, z*)	*t* value	Voxel number
Precentral _L	−36, −16, 58	4.88	66
Precentral _R	21, −16, 58	4.68	37
Postcentral _L	−51, −28, 52	5.78	20

### Correlation Analyses Between Altered Cerebral Blood Flow, Fractional Anisotropy, and Motor Function

**FIGURE 6:**
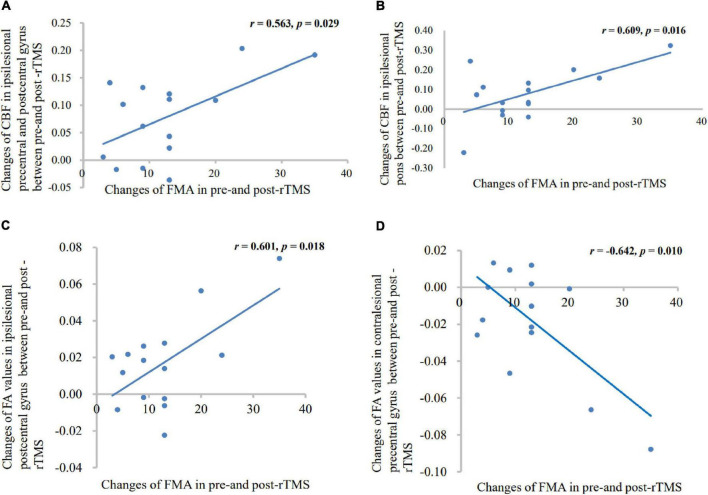
Correlation between the altered CBF **(A,B)**, FA **(C,D)** values, and motor scores changes. Patients with rTMS treatment displayed significant positive correlations between FMA score and altered CBF values in the ipsilesional precentral gyrus, postcentral gyrus, and pons; and showed significant positive correlations between FMA score and altered FA values in the ipsilesional postcentral gyrus; and a negative correlation between FMA score and altered FA value in the contralesional precentral gyrus.

Patients with rTMS treatment showed significant positive correlation between altered CBF of ipsilesional central anteroposterior gyrus (*p* = 0.029, *r* = 0.563), pons (*p* = 0.016, *r* = 0.609), and FMA difference. In addition, patients with rTMS treatment showed significant positive correlation between altered FA of ipsilesional postcentral gyrus and FMA difference (*p* = 0.018, *r* = 0.601), and negative correlation between altered FA of contralesional precentral gyrus and FMA difference (*p* = 0.010, *r* = −0.642), as shown in Figure 6.

## Discussion

In this study, we focused on the CBF and FA changes in stroke patients with rTMS intervention. Through the study, we found that decreased CBF was mainly detected in the ipsilesional corticocerebellar pathway in stroke patients; increased CBF occurred in the ipsilesional CPC pathway, while decreased in the contralesional hemisphere after rTMS treatment. And the FA value of ipsilesional CST significantly decreased after stroke, while increased after rTMS intervention.

Compared with the HCs, the CBF in the contralesional cerebellum and bilateral cerebral cortex decreased in stroke patients, with the occurrence of crossed cerebellar diaschisis (CCD) phenomenon. The phenomenon may be due to the inactivation of cerebellar neurons caused by the decrease of afferent excitation in CPC pathway (Dettmers et al., 1995; Gold and Lauritzen, 2002). An earlier research has reported that, whether supratentorial infarction or brainstem infarction, as long as it is located in CPC pathway, it can cause CCD (Tsuzuki et al., 1990). Another study also confirmed that the injury of CPC is the key to the occurrence of CCD (Fazekas et al., 1993). In addition, we found that the CBF was mainly improved in the ipsilesional CPC loop after rTMS treatment, and decreased in the contralesional hemisphere. Neurovascular coupling indicated that neuronal activity is related to CBF in spatial and temporal (Girouard and Iadecola, 2006). Increased neuronal activity increases brain metabolism, and then triggers hemodynamic response to increase CBF (Iyer and Madhavan, 2018). It can be inferred that after high-frequency rTMS treatment, neurogenesis and angiogenesis may occur in the injured pathway of the affected side, thus promoting the rehabilitation of motor function. The latest study of a rat photothrombotic stroke model showed that rTMS can significantly reduce the permeability of blood-brain barrier, improve vascular structure, morphology, and perfusion injury, promote and maintain angiogenesis after ischemia stroke for a long time (Zong et al., 2020). Besides, by comparing the effects of real rTMS and sham rTMS, we found that the CBF decreased in the contralesional hemisphere and increased in the ipsilesional hemisphere. The imbalance of CBF between hemispheres is closely related to the recovery of motor function after stroke (Wiest et al., 2014). There is a balanced inhibition between the two hemispheres in healthy brain. However, the inhibition of the affected side to the unaffected side is weakened after stroke, resulting in overexcitation of the unaffected side (Traversa et al., 1998). High-frequency rTMS could improve the excitability of the affected side, and the activity of motor cortex was closely related to the motor function (Du et al., 2019). To sum up, the CBF of the injured side increased while the uninjured side decreased after high-frequency rTMS treatment, which increased the inhibition of the injured side to the contralesional hemisphere, so as to reach the goal of hemispheric rebalance. But this is inconsistent with Khaleel et al.’s (2010) findings, who indicated that CBF velocity increased both in ipsilateral and contralateral in middle cerebral artery after high-frequency rTMS in acute ischemic stroke. The reasons for the difference may be as follows. First, we included patients with supratentorial and brainstem infarction. Second, the stimulation site of rTMS was different. Khaleel et al. (2010) stimulated the dorsolateral prefrontal cortex, while we stimulated the primary motor cortex. Third, the methods of measurement and evaluation were also different. We used ASL to assess the CBF, while Khaleel et al. (2010) measured the CBF velocity by transcranial Doppler.

The functional and structural changes between hemispheres both correlate with motor recovery (Wiest et al., 2014). In this study, in addition to the change of cerebral perfusion, the brain structure also changed in stroke patients. We found that the FA value of the CST in the affected side was significantly decreased. As reported in a previous study, FA decreased in all four levels with the pons, posterior limb of the internal capsule, corona radiata, and precentral gyrus in acute ischemic brainstem stroke (Chen et al., 2018). The CST is the most important fiber bundle related to movement. Growing evidence indicates that the integrity of CST is closely related to the recovery of motor function (Lindenberg et al., 2010), and the activation of motor cortex (Puig et al., 2017). Subsequently, we found increased FA values of the sensorimotor area, cerebellum in the affected side and that of SMA in the unaffected side; and decreased FA values in the contralesional motor area after rTMS treatment. There was a simple linear relationship between the activation of cerebellar hemisphere and motor recovery in better recovery patients (Small et al., 2002), just like our results showed. The FA values increased in both ipsilesional sensorimotor area and contralesional motor area by comparing with the sham rTMS. Activity in the unaffected hemisphere contributes to functional recovery after stroke, which is called vicariation mechanism (Di Pino et al., 2014). From the results of DTI, not only competition mechanism, but also vicariation mechanism existed between the two hemispheres after rTMS intervention, which is called a bimodal balance-recovery model (Di Pino et al., 2014; Di Pino and Di Lazzaro, 2020). This is inconsistent with the result of Li et al. (2018), who demonstrated that the FA increased in the contralesional corticocerebellar pathways might reflect the contralesional compensation by high-frequency rTMS. Furthermore, a study showed that the activation of the contralesional sensorimotor area was connected with the recovery of motor function (Small et al., 2002). These may be related to the degree of damage on the affected side. The more severe the damage is, the less structure reserves, and the greater activation of the contralesional hemisphere is. That is to say, vicariation mechanism is more prominent than competition mechanism under the circumstances (Di Pino et al., 2014). In general, the motor function recovery after stroke could be promoted through the microstructural plasticity modulated by rTMS on CST pathway in the affected side and vicariation mechanism of the unaffected side after rTMS intervention.

The CST forms one part of the CPC loop. Our results coincided with this, and also proved that structural changes would occur in the altered functional areas of the brain after rTMS intervention. In addition, combined with the results of CBF and FA before and after rTMS, we found that the areas with perfusion changes were more than those with structural changes, suggesting that functional change may be before the structural change. Maybe it is because microstructural plasticity takes a longer time to happen. It has been reported that mean FA values changed more significantly at 30 days than at admission and 3 days (Puig et al., 2010).

There was a significant positive correlation among the altered CBF of affected precentral gyrus, postcentral gyrus, and pons and FMA score changes in the rTMS group. The CPC tracts projected to the pons mainly by the corticopontine fibers from the precentral gyrus and postcentral gyrus (Voogd, 2003; Ramnani, 2006; Kamali et al., 2010), and crossed to the contralateral cerebellar peduncle. So, sensorimotor cortex and pons are two important nodes in CPC pathway. This positive correlation is convincing evidence of the efficacy of rTMS. In addition, these results suggest that the changes of CBF in the affected side are closely related to the motor recovery. A positive correlation was found between the altered FA values of affected postcentral gyrus and FMA score changes, and a negative relationship between unaffected precentral gyrus and altered FA values in the rTMS group, which exactly proved the process of brain rebalance.

There are some limitations in this study. First, the sample size we included was small, whether the results can be applied to more stroke patients still needs further study. Second, we did not make a follow-up with the patients, so the long-term efficacy of rTMS could not be evaluated objectively. Moreover, our design was not double-blind, so the evaluator probably existed subjective effects when assessing motor function. Finally, we did not make a more detailed classification of supratentorial and brainstem infarction. Future research needs to improve in these aspects.

## Conclusion

This study combined ASL and DTI to observe the cerebral perfusion and structure changes after the rTMS treatment and found some important regions, such as ipsilesional CPC loop and microstructure of CST which are closely related to motor functional reorganization.

## Data Availability Statement

The original contributions presented in the study are included in the article/Supplementary Material, further inquiries can be directed to the corresponding authors.

## Ethics Statement

The studies involving human participants were reviewed and approved by the Ethics Committee of the Nanchong Central Hospital. The patients/participants provided their written informed consent to participate in this study.

## Author Contributions

YJ designed the experiment. YJ and XB carried out the scale assessments, MRI data acquisition, and rTMS intervention jointly. YJ and BJ performed the statistical analysis. XB drafted the manuscript. ZG and QM reviewed and contributed to modifying the article. All authors approved the submitted version of the manuscript.

## Conflict of Interest

The authors declare that the research was conducted in the absence of any commercial or financial relationships that could be construed as a potential conflict of interest.

## Publisher’s Note

All claims expressed in this article are solely those of the authors and do not necessarily represent those of their affiliated organizations, or those of the publisher, the editors and the reviewers. Any product that may be evaluated in this article, or claim that may be made by its manufacturer, is not guaranteed or endorsed by the publisher.

## Footnotes

1
http://www.fil.ion.ucl.ac.uk/spm/

2
http://trackvis.org/
