# Supplementary material for: Repetitive Transcranial Magnetic Stimulation Induces Quantified Functional and Structural Changes in Subcortical Stroke: A Combined Arterial Spin Labeling Perfusion and Diffusion Tensor Imaging Study
Source: Front Hum Neurosci. 2022 Apr 6;16:829688. doi: 10.3389/fnhum.2022.829688 (PMC9019060; doi:10.3389/fnhum.2022.829688)
Supplement: Supplementary file 1 [file Data_Sheet_1.docx]

**Supplementary Material – Original data**

**Supp. Table 1. Information of the patient in high-frequency rTMS group**

| **NO.** | **Gender** | **Age** | **Foci** | **Duration** | **FMA** | | **NIHSS** | | **BI** | |
| --- | --- | --- | --- | --- | --- | --- | --- | --- | --- | --- |
|  |  |  |  |  | **Pre** | **Post** | **Pre** | **Post** | **Pre** | **Post** |
| 1 | M | 73 | L_BG | 2 | 30 | 34 | 9 | 7 | 30 | 40 |
| 2 | F | 56 | L_BG | 5 | 22 | 31 | 7 | 3 | 25 | 40 |
| 3 | F | 60 | R_BG | 4 | 40 | 64 | 3 | 3 | 50 | 80 |
| 4 | M | 53 | R_BG | 6 | 50 | 85 | 6 | 4 | 45 | 75 |
| 5 | M | 79 | L_BG | 6 | 12 | 18 | 9 | 7 | 30 | 45 |
| 6 | M | 66 | L_Pons | 5 | 34 | 47 | 8 | 3 | 55 | 85 |
| 7 | F | 72 | L_CR | 9 | 23 | 28 | 9 | 6 | 30 | 45 |
| 8 | F | 76 | R_BG | 6 | 21 | 34 | 7 | 3 | 45 | 65 |
| 9 | F | 48 | R_CR | 6 | 40 | 53 | 6 | 4 | 55 | 75 |
| 10 | M | 68 | L_BG | 5 | 56 | 69 | 6 | 4 | 60 | 80 |
| 11 | M | 62 | L_BG | 8 | 60 | 80 | 5 | 3 | 80 | 85 |
| 12 | M | 57 | R_Pons | 3 | 65 | 78 | 5 | 3 | 85 | 90 |
| 13 | M | 64 | L_CR | 2 | 19 | 28 | 9 | 7 | 30 | 40 |
| 14 | M | 76 | R_Pons | 4 | 25 | 34 | 9 | 7 | 45 | 55 |
| 15 | M | 57 | R_BG | 4 | 32 | 35 | 8 | 6 | 35 | 45 |

**Supp. Table 2. Information of the patient in sham rTMS group**

| **NO.** | **Gender** | **Age** | **Foci** | **Duration** | **FMA** | | **NIHSS** | | **BI** | |
| --- | --- | --- | --- | --- | --- | --- | --- | --- | --- | --- |
|  |  |  |  |  | **Pre** | **Post** | **Pre** | **Post** | **Pre** | **Post** |
| 1 | 63 | M | L_BG | 3 | 12 | 14 | 9 | 8 | 25 | 35 |
| 2 | 78 | F | L_CR | 6 | 25 | 26 | 7 | 7 | 35 | 50 |
| 3 | 60 | M | R_CR | 7 | 39 | 43 | 5 | 4 | 55 | 65 |
| 4 | 64 | M | L_BG | 2 | 56 | 59 | 6 | 5 | 40 | 65 |
| 5 | 64 | M | L_BG | 4 | 35 | 38 | 9 | 6 | 35 | 50 |
| 6 | 51 | F | L_Pons | 7 | 31 | 33 | 7 | 4 | 45 | 60 |
| 7 | 59 | F | L_CR | 2 | 22 | 23 | 8 | 8 | 25 | 35 |
| 8 | 61 | M | R_BG | 5 | 15 | 15 | 9 | 8 | 20 | 30 |
